# Supplementary material for: Health Outcomes Associated with Olive Oil Intake: An Umbrella Review of Meta-Analyses
Source: Foods. 2024 Aug 21;13(16):2619. doi: 10.3390/foods13162619 (PMC11353474; doi:10.3390/foods13162619)
Supplement: Supplementary file 1 [file foods-13-02619-s001.zip › foods-3126407-supplementary.pdf]

Supplementary Table S1. Item-by-item methodological quality of included meta-analyses

Selected articles	AMSTAR 2, ITEMS																QUALITY GRADE
	Q1	Q2	Q3	Q4	Q5	Q6	Q7	Q8	Q9	Q10	Q11	Q12	Q13	Q14	Q15	Q16	
Ke, 2024 (61)	YES	YES	N.A.*	YES	NO	YES	YES	YES	YES	N.A.	YES	NO	NO	YES	YES	YES	LOW
Morvaridzadeh, 2024 (47)	YES	YES	NO	YES	YES	YES	YES	YES	YES	NO	YES	YES	YES	YES	YES	YES	MODERATE
Tsamos, 2024 (48)	YES	YES	NO	YES	YES	YES	YES	YES	YES	NO	YES	YES	YES	YES	YES	YES	MODERATE
Armond Santos, 2023 (49)	YES	YES	NO	YES	YES	YES	YES	YES	YES	NO	YES	YES	YES	YES	YES	YES	MODERATE
Fakhri, 2023 (52)	YES	YES	YES	YES	YES	YES	NO	YES	YES	YES	YES	NO	NO	NO	NO	YES	CRITICALLY LOW
Jabbarzadeh-Ganjeh, 2023 (53)	YES	YES	YES	YES	YES	YES	YES	YES	YES	NO	YES	YES	YES	NO	NO	YES	CRITICALLY LOW
Zupo, 2023 (50)	YES	YES	YES	YES	YES	YES	NO	YES	YES	NO	NO	NO	YES	YES	NO	YES	CRITICALLY LOW
Hernández-Vásquez, 2022 (71)	YES	YES	YES	YES	YES	YES	YES	YES	YES	NO	YES	YES	YES	YES	NO (<10 PUBBL.)	YES	HIGH
Markellos, 2022 (62)	NO	YES	N.A.	YES	YES	YES	YES	YES	YES	N.A.	YES	NO	YES	YES	YES	YES	MODERATE
Martínez-Gonzalez, 2022 (4)	YES	YES	N.A.	YES	YES	YES	YES	YES	YES	N.A.	YES	NO	YES	YES	YES	YES	HIGH
Xia, 2022 (67)	NO	NO	N.A.	YES	NO	YES	YES	YES	YES	N.A.	YES	NO	NO	YES	YES	YES	CRITICALLY LOW
Dehghani, 2021 (55)	YES	NO	YES	YES	YES	YES	YES	YES	YES	NO	YES	NO	YES	YES	YES	YES	CRITICALLY LOW

Derakhshandeh-Rishehri, 2021 (51)	YES	YES	YES	YES	YES	YES	YES	YES	YES	NO	YES	YES	YES	YES	NO	YES	CRITICALLY LOW
Pastor, 2021 (54)	YES	YES	YES	YES	YES	YES	YES	YES	YES	NO	YES	YES	NO	NO	NO	YES	CRITICALLY LOW
Sealy, 2021 (63)	NO	NO	N.A.	YES	YES	YES	YES	YES	YES	N.A.	YES	NO	YES	YES	YES	YES	LOW
Fernandes, 2020 (14)	YES	YES	NO	YES	YES	YES	YES	YES	YES	NO	YES	YES	YES	NO	NO (<10 PUBBL.)	YES	MODERATE
Schwingshackl, 2019 (57)	YES	YES	YES	YES	NO	NO	YES	YES	YES	YES	YES	NO	NO	NO	NO	YES	CRITICALLY LOW
Tsartsou, 2019 (8)	YES	NO	YES	YES	YES	YES	YES	YES	NO	NO	YES	NO	NO	NO	NO	YES	CRITICALLY LOW
Eleftheriou, 2018 (72)	YES	YES	N.A.	NO	YES	YES	YES	YES	NO	N.A.	YES	NO	NO	NO	YES	YES	CRITICALLY LOW
George, 2018 (56)	YES	YES	YES	YES	YES	YES	YES	YES	YES	NO	YES	YES	YES	YES	NO	YES	CRITICALLY LOW
Ghobadi, 2018 (59)	YES	NO	YES	YES	YES	YES	YES	YES	YES	NO	YES	YES	YES	YES	YES	YES	CRITICALLY LOW
Zamora-Zamora, 2018 (58)	YES	NO	YES	YES	YES	YES	YES	YES	YES	NO	YES	YES	YES	YES	YES	NO	CRITICALLY LOW
Grosso, 2017 (68)	NO	NO	N.A.	YES	YES	NO	YES	YES	YES	N.A.	YES	NO	NO	YES	NO	NO	CRITICALLY LOW
Schwingshack, 2017 (7)	YES	YES	YES	YES	YES	YES	YES	YES	YES	NO	YES	YES	YES	YES	YES	YES	HIGH
Hohmann, 2015 (60)	YES	NO	YES	YES	YES	YES	YES	YES	YES	NO	YES	YES	YES	YES	YES	YES	MODERATE

Schwingshackl, 2015 (65)	YES	YES	NO	YES	YES	YES	YES	YES	YES	NO	YES	NO	NO	YES	YES	YES	CRITICALLY LOW
Xin, 2015 (70)	NO	NO	N.A.	YES	NO	NO	YES	YES	YES	N.A.	YES	YES	NO	YES	YES	YES	CRITICALLY LOW
Schwingshackl, 2014 (75)	NO	NO	N.A.	YES	YES	NO	NO	YES	YES	N.A.	YES	NO	NO	YES	YES	NO	CRITICALLY LOW
Martínez-Gonzalez 2014 (76)	NO	NO	N.A.	YES	NO	YES	YES	YES	YES	N.A.	YES	NO	YES	YES	YES	YES	CRITICALLY LOW
Pelucchi, 2011 (72)	NO	NO	N.A.	NO	NO	NO	NO	NO	NO	N.A.	YES	NO	YES	NO	NO	YES	CRITICALLY LOW
Psaltopoulou, 2011 (71)	NO	NO	N.A.	NO	NO	NO	NO	YES	NO	N.A.	YES	NO	NO	YES	NO	YES	CRITICALLY LOW

*Not available
